# The relationship among burden, anxiety, and depression among caregivers of elderly patients with chronic obstructive pulmonary disease: the chain-mediating effect of coping styles and social support

**DOI:** 10.3389/fpsyg.2026.1981977

**Published:** 2026-10-30

**Authors:** Jianghong Liu, Hongyan Lu, Haibo Ma, Yanan Deng, Ruijie Gu, Jiayu Liu, Lulu Qi

**Affiliations:** 1 School of Nursing, Ningxia Medical University, Yinchuan, Ningxia, China; 2 Department of Nursing, General Hospital of Ningxia Medical University, Yinchuan, Ningxia, China

**Keywords:** anxiety, burden, COPD, coping strategies, depression, informal caregivers, social support

## Abstract

**Purpose:**

Although anxiety and depression are common among caregivers of elderly patients with chronic obstructive pulmonary disease (COPD), previous studies have mostly examined caregiving burden, coping strategies, and social support separately, and studies on how these factors act jointly—particularly the serial pathway from burden to psychological distress—remain limited. Therefore, this study aimed to investigate whether negative coping strategies and social support serve as chain mediators in the relationship between caregiving burden and psychological distress, among primary caregivers of elderly patients with COPD.

**Methods:**

This was a cross-sectional study. A total of 240 primary caregivers of elderly patients with COPD, hospitalized in Ningxia, China, were recruited by convenience sampling between August 2025 and November 2025. Data were collected using a demographic questionnaire, the Zarit Caregivers Burden Interview (ZBI), the Hospital Anxiety and Depression Scale (HADS), the Simplified Coping Style Questionnaire–Negative Coping subscale (SCSQ-NCS), and the Social Support Rating Scale (SSRS). One-way analysis of variance (ANOVA) and Pearson correlation analyses were performed, and the chain mediation effects were estimated using the bootstrap method to test the proposed model.

**Results:**

Among the 240 caregivers, the mean age was 53.13 years and 60.8% were female; the mean caregiving burden score was 71.00 (standard deviation [SD] = 11.70), and the mean total HADS score was 22.18 (SD = 4.45). Age, education, relationship with the patient, occupation, monthly income, number of chronic diseases, sleep quality, and health status were the main factors associated with caregivers’ anxiety and depression scores. Anxiety and depression were positively correlated with caregiving burden and negative coping styles. The model results showed that caregiving burden directly and positively predicted anxiety and depression, and also indirectly predicted anxiety and depression through the chain mediation of negative coping styles and social support.

**Conclusion:**

Caregivers of elderly patients with COPD experienced a considerable caregiving burden along with anxiety and depression. Caregiving burden was directly associated with anxiety and depression and was also indirectly associated with them through serial mediation by negative coping styles and social support. These findings suggest that interventions to reduce maladaptive coping and strengthen perceived social support may help alleviate the psychological distress of these caregivers. Given that health literacy and care-related knowledge are generally limited among caregivers of patients with COPD, the future studies should examine whether such interventions remain effective when caregivers’ health literacy is low.

## 1 Introduction

Chronic obstructive pulmonary disease (COPD) is a progressive respiratory disease characterized by persistent respiratory symptoms and airflow limitation (Christenson et al., 2022). Its high prevalence and mortality rates make it a major global public health concern (Halpin, 2024). The global prevalence of COPD is estimated at 11.7%, while in China nearly 100 million people are affected, with a prevalence of 13.7% among individuals aged 40 years and older (Viegi et al., 2020). COPD is currently the third leading cause of death worldwide, accounting for approximately 3 million deaths annually (Celli et al., 2022). As the population continues to age, the number of people aged 60 years and older in China has exceeded 300 million (Wang A.-Q. et al., 2025). In this context, surveys indicate that the prevalence of COPD is 18.1% among those aged 60–69 years and as high as 32.3% among those aged 70 years and higher (Yu et al., 2024).

Older patients with COPD often present with comorbidities and age-related factors, such as cognitive decline, which pose substantial challenges to their independent disease management (Nielsen et al., 2025). For instance, neglecting early symptoms or using an inhaler incorrectly often reflects inadequate self-management, which can lead to frequent acute exacerbations, recurrent hospitalizations, and potentially respiratory failure (Barnestein-Fonseca et al., 2023). Evidence suggests that caregiver involvement supports more effective disease self-management among older adults with COPD (Ora et al., 2024). In this study, family caregivers are defined as relatives who provide unpaid, ongoing assistance with disease management for an older patient with COPD. Despite the reliance placed on them, family caregivers in China remain largely unrecognized by the health system, and specific support policies remain limited (Xu et al., 2025). Providing such assistance places sustained demands on the caregiver. These demands are conceptualized as caregiver burden, first defined by Zarit et al. (1980) as caregivers’ perceived suffering in health, social life, and finances, and grounded in appraisal theory (Lazarus and Folkman, 1984). In the caregiving stress process model, burden is a dynamic outcome shaped by primary caregiving stressors, secondary stressors that spill over into other life domains, and the buffering effects of coping and social support (Pearlin et al., 1990). Following this model, the present study conceptualizes caregiver burden as the caregiver’s subjective appraisal of the patient’s changing needs and the shifting caregiving context over time. Burden is common in COPD caregiving: in one study, 88% of caregivers reported moderate-to-severe burden (Jiao, 2022). This prolonged caregiving burden frequently leads to adverse psychological outcomes in caregivers, including anxiety, depression, and hopelessness. Previous research has shown that psychological symptoms, particularly anxiety and depression, are significantly more prevalent among family caregivers of COPD patients than in the general population (Gholami et al., 2025). Anxiety and depression are among the most prevalent mental health conditions worldwide. According to the World Health Organization, anxiety and depressive disorders are characterized by excessive worry and depressed mood along with related behavioral disturbances, and by symptoms severe enough to cause significant distress or impairment in personal, family, social, educational, occupational, or other important areas of functioning (American Psychiatric Association, 2013; World Health Organization, 2022). Unlike ordinary mood fluctuations, these conditions are clinically significant mental health problems that impair caregivers’ daily functioning (Malhi and Mann, 2018). Research confirms that these symptoms are closely linked to caregiving burden. Furthermore, untreated distress may compromise the quality of caregiving and the patient’s recovery, while jeopardizing the caregiver’s wellbeing (Rashid et al., 2023).

Coping strategies are defined as the cognitive and behavioral efforts individuals use in response to stressors, and are conventionally classified as positive or negative (Zhang et al., 2025). Positive coping strategies among caregivers can effectively buffer stress and sustain psychological wellbeing. In contrast, negative coping strategies tend to exacerbate burden and stress, thereby compromising both physical and mental health (Han et al., 2014). Longitudinal evidence suggests that prolonged reliance on maladaptive coping strategies, without timely modification, may seriously impede caregivers’ adaptation, further intensifying their caregiving burden and psychological distress (Rebolo et al., 2026). Notably, negative coping strategies are more readily identifiable and modifiable than positive ones (Muñoz-Cruz et al., 2024) because they manifest more explicitly in the caregiving context, thereby facilitating early detection and intervention (Zhang et al., 2025).

Social support, a critical external resource, is widely recognized as a key protective factor that alleviates caregiver burden and reduces psychological distress, including anxiety and depression (Cenkçi et al., 2024; Nasreen et al., 2024). Social support, defined as the assistance caregivers derive from social networks, mitigates stress, reduces psychological symptoms, and promotes social adjustment. Perceived social support is negatively associated with caregiver burden (Del-Pino-Casado et al., 2018) and with caregivers’ anxiety and depression (Del-Pino-Casado et al., 2021). It also mediates the relationship between caregiving burden and psychological distress (Graven et al., 2020).

Caregiver burden is the primary stressor in the caregiving process (Pearlin et al., 1990), and part of its effect on anxiety and depression is direct, while another part is transmitted through intervening variables. Guided by the caregiving stress process model, this study proposes that coping style and social support mediate this effect sequentially rather than in parallel. First, in the transactional model of stress and coping, coping is the caregiver’s proximal response to a stressor (Lazarus and Folkman, 1984; Park et al., 2021), whereas social support is an external protective resource (Muñoz-Cruz et al., 2023). On this account, a proximal response precedes the mobilization of an external resource. Second, internal coping resources shape the extent to which caregivers can use external resources. Consistent with this reasoning, a study of caregivers of older adults with dementia found that internal resources (coping strategies) and external resources (social support) served as mediators in the chain linking family burden and psychological distress (Wang et al., 2024). We hypothesize a serial mediation model in which caregiving burden is associated with anxiety and depression through negative coping strategies, which in turn lead to reduced social support.

Previous studies of caregivers of patients with COPD have focused predominantly on the burden–quality-of-life relationship, in which social support and negative coping strategies act as sequential mediators (Yi et al., 2021). Quality of life, however, is a broad outcome that may obscure the specific effects of caregiving burden on mental health, and whether the same sequential pathway also explains anxiety and depression has not been tested. Clarifying this pathway is practically relevant because it indicates whether interventions should target coping strategies, social support, or both. Accordingly, this study examines whether negative coping strategies and social support serve as mediators in the chain linking caregiving burden to anxiety and depression among family caregivers of older patients with COPD. We hypothesize that (H1) caregiving burden is positively associated with anxiety and depression; (H2) negative coping strategies and social support serially mediate this association; and (H3) the serial indirect effect is significant, such that higher caregiving burden is associated with greater reliance on negative coping strategies, lower perceived social support, and, consequently, higher levels of anxiety and depression.

## 2 Materials and methods

### 2.1 Study design, setting, and participants

This cross-sectional study is reported in accordance with the Strengthening the Reporting of Observational Studies in Epidemiology (STROBE) statement. The study was conducted in four hospitals in Ningxia, China, between August 2025 and November 2025, in compliance with the Declaration of Helsinki. The study protocol was approved by the Ethics Committee of the General Hospital of Ningxia Medical University (Approval No. KYLL-2025-2430). All participants provided written informed consent prior to participation.

Participants were recruited through convenience sampling. A primary family caregiver was defined as the relative who provided unpaid, ongoing assistance with disease management for an older patient with COPD. Caregivers were eligible if they were family members aged 18 years or older who assumed primary caregiving responsibilities; had provided care for more than 3 months; and possessed basic literacy in Chinese, normal comprehension and expression abilities, and intact communication skills. Caregivers were excluded if they had severe mental illness.

Sample size was determined based on the recommendations of Fritz and MacKinnon (2007). This approach corresponds to the analytic strategy used in the present study (the Statistical Package for the Social Sciences [SPSS] PROCESS macro with bootstrap estimation), rather than to structural equation modeling, which indicates that at least 200 participants are required to detect small-to-medium mediation effects with adequate statistical power. We therefore aimed to recruit at least 200 caregivers; ultimately, 240 were included in the analysis.

The scale of the problem in this setting is considerable. The Ningxia Hui Autonomous Region has a population of approximately 7.32 million, of whom about 1.21 million are aged 60 years or older (Department of Civil Affairs of Ningxia Hui Autonomous Region, 2026). Based on a reported COPD prevalence of 27% among older adults in China, approximately 330,000 older adults in Ningxia are estimated to have COPD, according to the on-site investigation: a 1:1 caregiver-to-patient ratio for patients requiring support, supported by approximately 330,000 family caregivers (Wang et al., 2018). To date, no registry-based data on caregivers of COPD patients are available in this region.

### 2.2 Instruments

The survey instrument comprised five sections: a general information questionnaire, the Zarit Burden Interview (ZBI), the Simplified Coping Style Questionnaire (SCSQ) negative coping subscale (SCSQ-NCS), the Social Support Rating Scale (SSRS), and the Hospital Anxiety and Depression Scale (HADS), for a total of 62 items. Participants completed the sections in the order presented below, and the questionnaire took approximately 20 min to complete.

#### 2.2.1 General information questionnaire

This questionnaire was developed by the research team based on the study objectives and content, informed by a literature review and team discussions. Caregiver fundamental information includes: gender, education, relationship with patients, occupation, monthly income, number of chronic diseases suffered, sleep quality, and health status.

#### 2.2.2 Zarit burden interview

The ZBI was developed by Zarit et al. (1986) and later translated into Chinese and culturally adapted by Wang et al. (2006). It comprises 22 items across two dimensions (personal burden and role burden). Each item is rated on a 5-point Likert scale ranging from 0 (never) to 4 (always), yielding a total score of 0–88, with higher scores indicating a greater caregiver burden. According to the conventional severity classification, a total score of 0–20 indicates little or no burden, 21–40 indicates mild-to-moderate burden, 41–60 indicates moderate-to-severe burden, and 61–88 indicates severe burden. The scale has previously been used to measure burden among caregivers, with a reported Cronbach’s α of 0.948 (Al Ghammari et al., 2026). In the present study, the Cronbach’s α coefficient was 0.913.

#### 2.2.3 Simplified coping style questionnaire of negative coping

The SCSQ was developed by Xie (1998). It contains 20 items across two dimensions, positive coping (items 1–12) and negative coping (items 13–20), each rated on a Likert scale 0–3. Only the negative coping subscale (SCSQ-NCS) was used in the present study to capture the degree of negative coping among caregivers. Higher scores on this subscale indicate a greater reliance on negative coping strategies. The subscale has previously been used to measure negative coping, with a reported Cronbach’s α of 0.806 (Yi et al., 2021). In the present study, the Cronbach’s α coefficient was 0.732.

#### 2.2.4 Social support rating scale

The SSRS was developed by Xiao (1994) and comprises 10 items across three dimensions: objective support (possible range: 1–22), subjective support (8–32), and utilization of social support (3–12), yielding a total score of 12–66. Higher scores indicate greater perceived social support; a total score below 22 was classified as low, 22–44 as moderate, and 44 or above as high. The scale has previously been used to measure social support, with a reported Cronbach’s α of 0.869 (Li et al., 2026). In the present study, the Cronbach’s α coefficient was 0.791.

#### 2.2.5 Hospital anxiety and depression scale

The HADS was developed by Zigmond and Snaith (1983) and was administered in its validated Chinese version (Lee et al., 2017). It comprises 14 items, with seven assessing anxiety (HADS-A) and seven assessing depression (HADS-D). Each item is scored from 0 to 3, yielding subscale scores of 0–21 and a total score of 0–42, with higher scores indicating more severe symptoms. In the present study, the total HADS score (HADS-T) was used as an index of overall psychological distress. The use of the HADS-T as a measure of general distress is supported by confirmatory factor analyses showing that, in addition to their specific components, the two subscales load onto a higher order distress dimension (Martínez López et al., 2012). For descriptive purposes, a score of ≥7 on either subscale was used to indicate the presence of clinically significant anxiety or depressive symptoms, and participants scoring ≥7 on both subscales were classified as having comorbid anxiety and depression. The scale has previously been used to assess anxiety and depression, with a reported Cronbach’s α of 0.850 (Agasha et al., 2026). In the present study, Cronbach’s α for the total scale was 0.879.

### 2.3 Data collection

Researchers administered the survey in person to participants in person using standardized instructions. After providing written informed consent, caregivers completed the questionnaire independently. Caregivers who had difficulty completing the questionnaire provided oral responses, which the researchers recorded and transcribed verbatim. All completed questionnaires were reviewed on site, and any ambiguities were clarified with the participant immediately. A total of 250 questionnaires were distributed, of which 240 were valid, yielding a response rate of 96%.

### 2.4 Data analysis

The statistical analysis was performed using IBM SPSS version 27. The analysis proceeded in the following order. Data screening: The dataset was first examined for completeness. No missing values were identified; therefore, neither data imputation nor case-wise deletion was required. Descriptive statistics: Normality was assessed using the Shapiro–Wilk test together with visual inspection of histograms and Q–Q plots. Continuous variables were expressed as mean ± standard deviation (SD) when normally distributed and as median and interquartile range (IQR) when not normally distributed. In contrast, categorical variables were reported as frequencies and percentages [n (%); *n* = number of cases]. Group comparisons and correlation analyses: Group differences in the study variables were examined using independent samples *t*-tests, Mann–Whitney U tests, one-way analysis of variance (ANOVA), or Kruskal–Wallis H tests, as appropriate; Correlations among caregiving burden, negative coping, social support, and anxiety and depression were examined using Pearson correlation coefficients for normally distributed variables and Spearman correlation coefficients when the normality assumption was not satisfied. Assessment of common method bias and multicollinearity: Common method bias was assessed using Harman’s single-factor test: 14 factors had eigenvalues greater than 1, and the first factor accounted for 23.12% of the total variance, below the 40% threshold, indicating no serious common method bias. Multicollinearity was assessed using variance inflation factors (VIFs); all were below 2.5, indicating no multicollinearity. Chain mediation analysis. A chain mediation model was constructed using the PROCESS macro version 4.2 for SPSS (Model 6). Indirect effects were estimated using 5,000 bootstrap samples, and 95% confidence intervals (CIs) were calculated. Variables that were statistically significant in the group comparisons were included as covariates in the mediation model. Statistical significance was set at *p* < 0.05.

## 3 Results

### 3.1 Participant characteristics

We collected data from 240 caregivers accompanying COPD patients during their hospitalization. In the study, the age range of caregivers was between 24 and 85 years; the female proportion was 60.8%; the majority of them had an educational attainment of primary school level or below(45.8%), the majority were the patients’ children (77.1%), and the majority of them provided full-time care at home (29.2%); see Table 1 for further details. The results showed that the care burden score for the primary caregivers of COPD patients was 71.00 (SD = 11.70), while their overall anxiety and depression score was 22.18 (SD = 4.45). The score for negative coping strategies was 12.20 (SD = 3.49), and the score for social support was 42.36 (SD = 6.79); see Table 2 for further details.

**Table 1:** Comparison of psychological distress (total HADS score) among caregivers with different demographic characteristics.

Category	Subcategory	N (%)	Anxiety and depression
Age	18–44	41(17.1%)	21.83 ± 4.68
	45–59	138(57.5%)	21.74 ± 4.33
	≥60	61(25.4%)	23.44 ± 4.41
*F*			3.319
*P*			0.038
Education	Primary school and below	111(46.3%)	23.18 ± 4.37
	Junior high school	63(26.3%)	21.33 ± 4.44
	Senior high school/secondary	32(13.3%)	22.26 ± 4.56
	Vocational school/junior college and above	34(14.1%)	22.19 ± 4.45
*F*			4.647
*P*			0.004
Relationship with patients	Spouses	45(18.8%)	24.33 ± 4.00
	Children	185(77.1%)	21.61 ± 4.40
	Siblings	3(1.2%)	20.67 ± 3.87
	Grandchildren	7(2.9%)	22.19 ± 4.45
*F*			5.544
*P*			0.001
Occupation	Unemployed	71(29.6%)	23.04 ± 4.11
	Retired or separated from employment	35(14.6%)	23.49 ± 4.19
	Employed in government agencies	45(18.8%)	21.07 ± 4.11
	Enterprises or institutions	53(22.1%)	22.64 ± 4.83
	Engaged in farming at home	34(14.2%)	19.47 ± 3.85
	Self-employed and Other	2(7%)	28.50 ± 4.45
*F*			5.632
*p*			<0.001
Income per month	<1,500	58(24.2%)	23.43 ± 4.50
	1,500–2,999	51(21.2%)	22.73 ± 4.26
	3,000–4,999	30(12.5%)	20.73 ± 3.87
	≥5,000	101(42.1%)	21.63 ± 4.52
*F*			3.446
*p*			0.017
Number of chronic diseases suffered	None	95(39.6%)	21.42 ± 4.52
	Yes	145(60.4%)	22.69 ± 4.35
*F*			4.372
*p*			0.031
Sleep quality	Very good	35(14.6%)	20.40 ± 4.25
	Good	91(37.9%)	21.85 ± 4.27
	Average	55(22.9%)	22.60 ± 4.30
	Poor	41(17.1%)	22.93 ± 4.82
	Very poor	18(7.5%)	24.44 ± 4.27
*F*			3.215
*p*			0.014
Health status	Very good	46(19.2%)	20.76 ± 4.16
	Good	91(37.9%)	21.76 ± 4.55
	Average	60(25.0%)	22.97 ± 4.52
	Poor	6(2.5%)	23.50 ± 3.89
	Very poor	37(15.4%)	23.54 ± 4.05
*F*			2.927
*p*			0.022

**Table 2:** Pearson correlations between the four variables of caregiving burden, social support, negative coping, and anxiety and depression.

Variable	MD ± SD	1	2	3	4
1. Caregiving burden	71.00 ± 11.70	1.000			
2. Anxiety and depression	22.18 ± 4.45	0.485**	1.000		
3. Negative coping	12.20 ± 3.49	0.349**	0.377**	1.000	
4. Social support	42.36 ± 6.79	−0.363**	−0.420**	−0.281**	1.000

*p < 0.05, **p < 0.01.

One-way ANOVA results showed that caregivers’ current anxiety and depression scores differed in terms of age, education, relationship with patients, occupation, income per month, number of chronic diseases suffered, sleep quality, health status, and patient gender were statistically significant.

### 3.2 Pearson correlation analysis

As shown in Table 2, Pearson correlation analysis showed a positive correlation between caregiving burden and anxiety and depression (*r* = 0.485, *p* < 0.01), and a negative correlation with negative coping style (*r* = 0.349, *p* < 0.01), and social support (*r* = −0.363, *p* < 0.01). Anxiety, depression, and social support were negatively correlated (*r* = −0.420, *p* < 0.05) and positively correlated with negative coping (*r* = 0.377, *p* < 0.01). The correlation coefficients indicated that the effect sizes between the variables ranged from−0.1 to 0.1, and the *p*-values for the correlations were statistically significant.

### 3.3 Analysis of chain mediation effects

The total effect of caregiving burden on anxiety and depression(c = 0.1763, *p* < 0.01) was statistically significant, and the direct effect of the path was(c = 0.1151, *p* < 0.01). Caregiving burden had a positive direct effect on negative coping (*β* = 0.258, *p* < 0.01) and a negative direct effect on social support (*β* = **−**0.153, *p* < 0.01). Also, negative coping style, as the first mediating variable, had a significant negative direct effect on social support, the second mediating variable (*β* = **−**0.337, *p* < 0.01), and the results of the chain mediation model analysis are shown in Figure 1.

**Figure 1:**
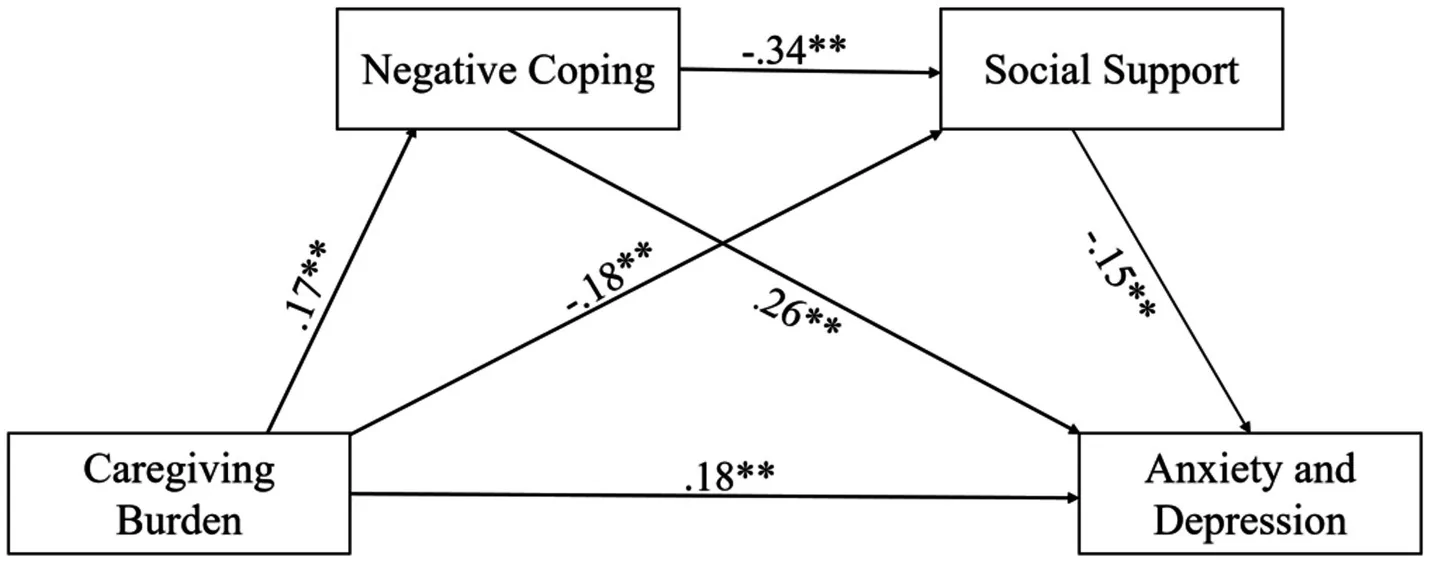
The role of negative coping and social support as chain mediators in the relationship between caregiving burden and anxiety and depression with standardized *β*. **p < 0.01. Values on paths are path coefficients (standardized *β*s).

As shown in Table 3, the paths through the single mediating variable M1 negative coping style (point estimate = 0.028; 95% bias-corrected confidence interval (BC CI): [0.010, 0.047]), the path through two mediators (point estimate = 0.006; 95% BC CI: [0.001, 0.013]), and the path through the single mediating variable M2 social support (point estimate = 0.028; 95% BC CI: [0.011, 0.047]) were all statistically significant. Moreover, the total indirect effect was statistically significant (point estimate = 0.061; 95% BC CI: [0.035, 0.087]). Thus, the path through these two chained mediating variables was significant, and the path through the indirect effects through negative coping style and social support alone were also significant.

**Table 3:** Chain-mediated effects of caregiving burden on anxiety and depression of caregivers of COPD patients through negative coping styles and social support.

Effect	Model	Point estimate	Effect value (%)	SE	95% CI	*p*
Total effect	X–Y	0.176	100.00	0.022	[0.133, 0.220]	<0.001
Direct effect	X–Y	0.115	65	0.023	[0.069, 0.161]	<0.001
Indirect effect	1	0.028	16	0.010	[0.010, 0.047]	<0.001
	2	0.028	16	0.009	[0.011, 0.047]	<0.001
	3	0.006	3.4	0.003	[0.001, 0.013]	<0.001

X = burden of care; M1 = negative coping; M2 = social support; Y = anxiety and depression. Model 1 = caregiving burden–negative coping–anxiety and depression. Model 2 = caregiving burden–social support–anxiety and depression. Model 3 = caregiving burden–negative coping–social support–anxiety and depression. SE, standard error.

Specifically, the mediation model operated via three pathways: (1) the pathway through negative coping strategies alone; (2) the pathway through social support alone; (3) the sequential pathway through negative coping strategies followed by social support. All three indirect effects were statistically significant, underscoring the importance of coping strategies and social support in alleviating anxiety and depression among caregivers of older adults with COPD.

## 4 Discussion

Caregivers of older patients with COPD in the present sample reported a severe burden (mean score 71.00), a level considerably higher than that reported in a multicenter Turkish sample (Baha et al., 2022) and in a French cohort of caregivers (Grosbois et al., 2022). A comparable pattern has been documented in China in a four-hospital sample of 201 caregivers; burden reached a severe level and exceeded that reported in Turkish and Portuguese samples (Yi et al., 2021). This difference is most likely attributable to the timing of recruitment, as participants were enrolled during or shortly after an acute exacerbation. Burden of this order has also been described in South Korea, where family caregivers of patients with COPD reported a considerably high level of strain (Kim et al., 2010). The level of burden is unlikely to reflect case mix alone, since the Turkish sample was also recruited in hospital and included a comparable proportion of patients with severe airflow limitation (Baha et al., 2022). A more plausible source of the difference lies in the structure of care surrounding the patient. In China, long-term and respite services remain limited (Zhang and Harper, 2022), and after being discharged from the hospital, patients usually return to their families, so responsibilities that elsewhere might be shared among formal and informal providers tend to fall on one or two relatives. Consistent with this, the burden among caregivers of patients with COPD in China has been reported to exceed that among Chinese caregivers of patients with stroke, a condition that is likewise disabling and long term (mean ZBI 30.43; Wang C. et al., 2025).

The mean total HADS score in the present sample was 22.18 (SD = 4.45), exceeding the cut-off of 15 commonly used to indicate clinically significant emotional distress on the HADS total score and exceeding the levels reported in previous studies of COPD caregivers (Liu et al., 2025; Grosbois et al., 2022). The severity of anxiety and depression, however, is likely to arise more directly from what the illness asks of caregivers over time and sustained care burden. As the disease progresses, elderly patients with COPD experience frequent acute exacerbations—a potentially traumatic event for caregivers (Cai et al., 2025), yet their significance may lie less in any single episode than in the interval that follows: because the next deterioration cannot be anticipated with certainty, caregivers tend to remain watchful, a state that only rarely subsides when the patient stabilizes. Distress of this kind is likely to accumulate rather than resolve between crises, which accords with the accounts of family members who describe isolation, loneliness and depression (Lindqvist et al., 2013), and of those who describe themselves as confined at home, with shrinking networks and persistent fear (Strang et al., 2018).

This study’s mediation findings are consistent with the caregiving stress process model (Pearlin et al., 1990), which indicates that reducing negative coping strategies and strengthening social support could effectively alleviate caregiving burden and mitigate psychological distress, and are perhaps most informative when they run counter to intuition. Negative coping is often interpreted as a deficit in the caregiver, yet it may be better understood as a response that is adaptive in the short term but costly over the long term: avoidance, self-blame, and venting relieve immediate tension while withdrawing the caregiver from the very interactions through which support is obtained. The pathway from burden through negative coping and diminished support to distress may therefore describe not two independent risk factors but a single process in which coping erodes the resource that might have offset it. Social support appears to be protective against the effects of negative coping (Zhang et al., 2024) and provides a setting in which emotion can be expressed rather than suppressed (Fang et al., 2024).

Cultural expectations are likely to compound this distress by shaping what caregivers feel able to do with it. China, Japan, and South Korea share broad similarities in cultural traditions, economic development models and population structures (Wang Y. et al., 2025), and caregiving in East Asian settings is commonly understood as a prescribed family duty rather than a role one chooses. When care is construed as an obligation, it is typically harder to decline, delegate, or negotiate, and outside help is perhaps less readily sought; those least able to reduce their exposure to the stressor may therefore also be the least likely to draw on the support that buffers them. Individual circumstance points in the same direction: lower household income and poorer self-rated health were associated with more anxiety and depression, in keeping with evidence that caregivers with fewer resources experience more persistent negative affect (Sami et al., 2025).

These findings point to two entry points for support. Structured education and care training has been reported to strengthen caregivers’ sense of competence and lighten care-related strain (Zhou et al., 2026), and scheduled psychological support—follow-up contact or telephone-based counseling—has been reported to relieve distress (Sami et al., 2025). Because negative coping and diminished support appear to lie on the same pathway, the two would seem to work better together than apart; this inference follows from the model tested here rather than from a direct trial.

## 5 Strength and limitations

A key strength of this study is its inclusion of participants from hospitals at different tiers, which enhances sample diversity and allowed us to examine both the direct and indirect effects within the proposed chain mediation model. However, several limitations should be acknowledged. First, the sample was drawn exclusively from the Ningxia Hui Autonomous Region in China, which may limit the generalizability of the findings. The future research could incorporate multi-regional samples to improve external validity. Second, the cross-sectional design precludes causal inferences regarding the relationships among caregiving burden, negative coping, social support, and psychological distress (anxiety and depression). Longitudinal studies are therefore warranted to clarify the temporal dynamics among these variables. Finally, the research did not account for potential confounding effects of researcher-provided counseling and guidance during data collection.

## 6 Conclusion

This study found that caregivers of older patients with COPD experience a substantial caregiving burden, which is positively correlated with their anxiety and depression. The chain mediation analysis further indicated that reducing negative coping strategies and strengthening social support could effectively alleviate the caregiving burden and mitigate psychological distress. These findings suggest that healthcare professionals should routinely assess caregivers’ burden and provide targeted support to strengthen their coping capacity. The future longitudinal and interventional studies are needed to test whether interventions that target negative coping and social support can reduce caregiver burden and psychological distress in this population. Moreover, replicating this study in diverse cultural and geographical contexts, particularly those experiencing rapid demographic aging and a high prevalence of older adults with COPD, would help determine the generalizability of these findings.

## Data Availability

The original contributions presented in the study are included in the article/supplementary material, further inquiries can be directed to the corresponding author.
